# A New Machairodont from the Palmetto Fauna (Early Pliocene) of Florida, with Comments on the Origin of the Smilodontini (Mammalia, Carnivora, Felidae)

**DOI:** 10.1371/journal.pone.0056173

**Published:** 2013-03-13

**Authors:** Steven C. Wallace, Richard C. Hulbert

**Affiliations:** 1 Department of Geosciences, Don Sundquist Center of Excellence in Paleontology, East Tennessee State University, Johnson City, Tennessee, United States of America; 2 Florida Museum of Natural History, University of Florida, Gainesville, Florida, United States of America; Durham University, United Kingdom

## Abstract

South-central Florida’s latest Hemphillian Palmetto Fauna includes two machairodontine felids, the lion-sized *Machairodus coloradensis* and a smaller, jaguar-sized species, initially referred to *Megantereon hesperus* based on a single, relatively incomplete mandible. This made the latter the oldest record of *Megantereon*, suggesting a New World origin of the genus. Subsequent workers variously accepted or rejected this identification and biogeographic scenario. Fortunately, new material, which preserves previously unknown characters, is now known for the smaller taxon. The most parsimonious results of a phylogenetic analysis using 37 cranio-mandibular characters from 13 taxa place it in the Smilodontini, like the original study; however, as the sister-taxon to *Megantereon* and *Smilodon*. Accordingly, we formally describe *Rhizosmilodon fiteae* gen. et sp. nov. *Rhizosmilodon*, *Megantereon*, and *Smilodon* ( =  Smilodontini) share synapomorphies relative to their sister-taxon Machairodontini: serrations smaller and restricted to canines; offset of P3 with P4 and p4 with m1; complete verticalization of mandibular symphysis; m1 shortened and robust with widest point anterior to notch; and extreme posterior “lean” to p3/p4. *Rhizosmilodon* has small anterior and posterior accessory cusps on p4, a relatively large lower canine, and small, non-procumbent lower incisors; all more primitive states than in *Megantereon* and *Smilodon*. The former also differs from *Megantereon* and *Smilodon gracilis* by having a very small mandibular flange. *Rhizosmilodon* is the oldest known member of the Smilodontini, suggesting that the tribe originated in North America. Two more derived, similar-sized species evolved in parallel during the Blancan, *Megantereon hesperus* and *Smilodon gracilis*. The former is rarer, known only from the north-central and northwestern US, and presumably dispersed into the Old World. The latter is known from the eastern and southern US, and dispersed into South America.

## Introduction

Most vertebrate fossils from the Upper Bone Valley Formation of Central Florida are recovered as isolated finds, either *in situ* from exposures created by mining operations or out of stratigraphic context in spoil piles [1]–[6]. Rarely are they found in sufficient concentration to allow quarrying. However, the largest known such concentration was discovered in the spring of 1989 in the Fort Meade Mine of Gardinier Inc. Thousands of vertebrate fossils were found in a single bed about 0.8 m thick that covered an area of about 2,000 m^2^. This assemblage, called the Whidden Creek Local Fauna, includes 33 mammalian taxa, of which 11 are carnivorans (Table 1). However, carnivorans account for only about 5% of the ca. 900 identifiable mammalian fossils from the Whidden Creek LF, in contrast to proportionally greater representation by perissodactyls (∼33%), artiodactyls (∼37%), and cetaceans (∼16%). The composite vertebrate fossil assemblage from the Upper Bone Valley Formation is referred to as the Palmetto Fauna [3], [6].

**Table 1:** Whidden Creek Local Fauna, latest Hemphillian, Polk County, Florida (UF locality PO054).

*Pristis* sp.	*Phalacrocorax* sp.
*Rhynchobatus* sp.	*Australca* sp.
*Dasyatis* sp.	*Diomedia sp.*
*Aetobatus narinari*	*Megalonyx curvidens*
*Myliobatus* sp.	*Borophagus hilli*
*Rhinoptera bonasus*	*Lynx rexroadensis*
*Carcharias taurus*	*Machairodus coloradensis*
*Hemipristis serra*	*Rhizosmilodon fiteae* gen. et sp. nov.
*Carcharocles megalodon*	cf. *Martinogale* sp.
*Negaprion brevirostris*	*Enhydritherium terraenovae*
*Carcharhinus* sp.	*Ontocetus emmonsi*
*Rhizoprionodon terrranovae*	Phocidae, genus and sp. indet.
*Galeocerdo contortus*	*Arctonasua eurybates*
*Galeocerdo cuvier*	*Agriotherium schneideri*
*Acipenser* sp.	*Plionarctos* sp.
*Lepisosteus* sp.	*Catagonus brachydontus*
*Centropomus* sp.	*Pleiolama vera*
*Caranx* sp.	*Megatylopus gigas*
*Archosargus probatocephalus*	*Hemiachenia edensis*
*Lagodon rhomboides*	*Floridameryx floridanus*
*Pogonias cromis*	*Eocoileus gentryorum*
*Sparisoma* sp.	*Hexameryx simpsoni*
*Sphyraena barracuda*	*Goniodelphis hudsoni*
*Balistes* sp.	*Balaenoptera floridana*
*Diodon* sp.	*Balaenoptera* sp.
*Macroclemys* sp.	*Nannippus aztecus*
*Apalone ferox*	*Cormohipparion emsliei*
*Trachemys inflata*	*Neohipparion eurystyle*
*Terrapene* sp.	*Dinohippus mexicanus*
*Gopherus* sp.	*Tapirus polkensis*
*Hesperotestudo* sp.	*Tapirus* sp.
*Hesperotestudo hayi*	*Teleoceras hicksi*
*Caretta* sp.	*Gomphotherium simplicidens*
*Chelonia* sp.	*Rhynchotherium edense*
*Alligator* sp.	*Mammut matthewi*

Marine and terrestrial vertebrates were recovered intermingled in the same stratigraphic horizon. Mammalian taxonomy after [6].

Among the carnivorans recovered from the Palmetto Fauna are the remains of a jaguar-sized machairodont cat [6]. Berta and Galiano [7] originally identified this felid as *Megantereon hesperus* based on a single partial ramus containing p3, p4 and the anterior portion of m1, making it the earliest record of the widespread genus *Megantereon*, which suggests a New World origin for this machairodont. While their identification as *Megantereon* was followed by some workers [8], [9], it was questioned by others due to the fragmentary nature of the material [6], [10], [11], [12], and more recent work even suggested that it exhibited close affinities to a specimen identified as “*Paramachairodus*” sp. from Arizona [13]. Just as important, is the Palmetto machairodont’s relationship to other members of the tribe Smilodontini, including *Smilodon*. Primarily because though *Smilodon* is an iconic member of the Pliocene and Pleistocene American faunas, its highly derived state has made interpreting its origin, and relationship to other closely related taxa, problematic [7], [9]–[12], [14]–[19].

**Figure 1:**
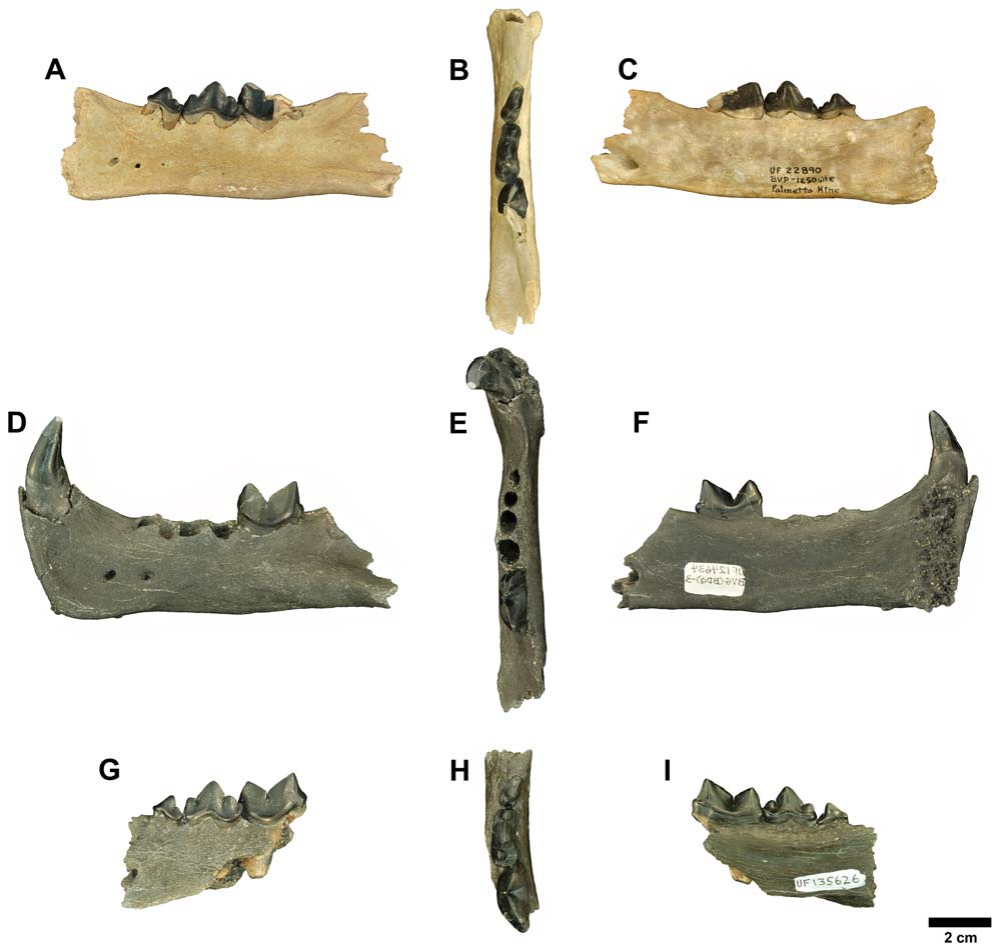
Examples of the Palmeto Fauna machairodont. Original described specimen UF 22890 [7] (A–C), proposed holotype UF 124634 (D–F), and paratype UF 135626 (G–I) in lateral, occlusal, and lingual views respectively. Images in D–F reversed to match the other two specimens.

More specifically, though *Megantereon* is well known from the Old World, its origins are uncertain [9], [ 11]. Several authors have suggested at least a sister relationship between *Megantereon* and *Smilodon* [7], [17], [18]; with some further suggesting that the former is ancestral to the latter [11], [15], [17]. In addition, there are suggestions that *Paramachaerodus* is a basal member of the Smilodontini [11], [13], [19], and that it gave rise to *Megantereon* [7], [10], [13], which in turn gave rise to *Smilodon* [11], [13], [15], [17]. Still, others have suggested an African origin for *Megantereon* [10] with a subsequent migration into the New World, thereby complicating the resolution of the relationships. However, if the Palmetto machairodont is indeed *Megantereon*, then a New World origin for at least *Megantereon* [9] would be supported. It is also possible that both *Megantereon* and *Smilodon* originated in North America [13]. Following several others [10], [12] who disagreed with the identification of the Palmetto machairodont and the suggestion that *Megantereon* dispersed from the Old World into the New, we sought to resolve some of these questions by reevaluating the former. Consequently, the recovery of additional specimens of the Palmetto machairodont (Figure 1), which provide characters not previously observed, affords the opportunity to revisit the systematic position of this taxon and to address some of the issues within the tribe.

## Methods

Basic taxonomy follows that of [11], [18], [19]. The acronym ‘UF’ refers to specimens cataloged into the vertebrate paleontology collection of the Florida Museum of Natural History, University of Florida, Gainesville.

Thirty-seven cranio-mandibular characters (Appendix S1) were scored on 13 taxa (*Proailurus lemanensis, Promegantereon ogygia, Paramachaerodus orientalis, P. maximiliani, Rhizosmilodon fiteae, Smilodon gracilis, S. fatalis, S. populator, Megantereon cultridens, M. hesperus, Machairodus aphanistus, M. coloradensis,* and *Homotherium serum*). Taxa were selected to represent the more derived subfamilies Machairodontini and Smilodontini, to include previous identifications of the Palmetto Fauna machairodont, and to build upon the recent revision (and spelling correction) of the genus *Paramachaerodus* [20]. Consequently, the first 25 characters are modified from that study. Supplemental characters reflect the additional features provided by the new material.

Cladistical analyses were run using PAUP 4.0 Beta 10 and MacClade. The most parsimonious (shortest) trees were evaluated using a heuristic search based on combined branch length.

*Nomenclatural Acts* – The electronic edition of this article conforms to the requirements of the amended International Code of Zoological Nomenclature, and hence the new names contained herein are available under that Code from the electronic edition of this article. This published work and the nomenclatural acts it contains have been registered in ZooBank, the online registration system for the ICZN. The ZooBank LSIDs (Life Science Identifiers) can be resolved and the associated information viewed through any standard web browser by appending the LSID to the prefix "http://zoobank.org/". The LSID for this publication is: urn:lsid:zoobank.org:pub: urn:lsid:zoobank.org:pub:41E89F59-8327-4282-8BC3-E3A195C436D2. The electronic edition of this work was published in a journal with an ISSN, and has been archived and is available from the following digital repositories: PubMed Central, LOCKSS.

## Results and Discussion

**Figure 2:**
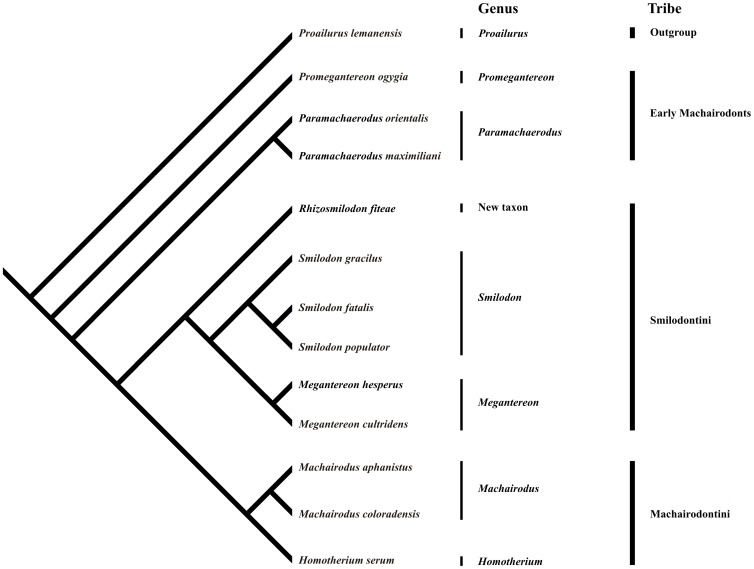
One of two most parsimonious trees based on 37 cranio-mandibular characters scored on 13 taxa (*Proailurus lemanensis, Promegantereon ogygia, Paramachaerodus orientalis, P. maximiliani, Rhizosmilodon fiteae, Smilodon gracilis, S. fatalis, S. populator, Megantereon cultridens, M. hesperus, Machairodus aphanistus, M. coloradensis,* and *Homotherium serum*). Tribes (specifically use of Machairodontini instead of the more familiar Homotherini) follow McKenna and Bell [29], in accordance with ICZN article 36 (Principle of Coordination). Thus, Gill [30] not only established the subfamily Machairodontinae, but also the tribe Machairodontini, and this name has priority over Homotherini Fabrini, 1890. TL  =  89, CI  =  0.63, RI  =  0.74, and RC  =  0.47.

*Phylogeny* – Scores are listed in Table 2. Results (Figure 2) agree with the revision of the late Miocene felid *Paramachaerodus* [20], with *Promegantereon* falling out as a distinct genus (from the former), and both falling basal to all other machairodonts. Of the trees evaluated, 2 most parsimonious trees were retained (Figure 2, Appendix S2). The first most parsimonious tree used in this study had: TL  =  89, CI  =  0.63, RI  =  0.74, and RC  =  0.47. A second most parsimonious tree is provided in Appendix S2.

**Table 2:** Character matrix

	1111111111222222222233333333
	1234567890123456789012345678901234567
*Proailurus lemanensis*	x000000000000000000000000000000000000
*Promegantereon ogygia*	0010100000000010010200200010010200010
*Paramachaerodus orientalis*	10121011110101101111112000101x0200010
*Paramachaerodus maximiliani*	1012101110x01110111xx1xxx0x01x02000x0
*Rhizosmilodon fiteae*	x0111000x1xxxxxxxxx2112020111x2201022
*Smilodon gracilus*	1011211111111110111211202111122301022
*Smilodon fatalis*	10122121111102111112112231111223110x2
*Smilodon populator*	10122121111102111112112231111223110x2
*Megantereon cultridens*	10102111110001100102212021112222x1022
*Megantereon hesperus*	xx1x20x0xxxxxxxxxxx221xx2x112x2xx10xx
*Machairodus aphanistus*	2113112110111110010111111100011200100
*Homotherium serum*	2113112110010211011111202100121100111
*Machairodus coloradensis*	2113112110111110110111111100121100111

Spelling of *Paramachaerodus* follows Salesa et al [20], in which a history of the spelling justifies the correction. Note that “x” indicates that the character was either not preserved, or in some way unable to be scored. Underlined scores indicate that the feature was scored based on other criteria, but may not have been directly observed (for example, presence of a mandibular flange indicates that the upper canine is elongated and flat, even if not actually preserved). A full list of specimens utilized in this study is available upon request.

### Systematic Paleontology

Order Carnivora Bowdich 1821

Family Felidae Fischer de Waldheim 1817

Subfamily Machairodontinae Gill 1872

Tribe Smilodontini Kretzoi 1929

*Rhizosmilodon fiteae* gen. et. sp. nov.

#### Etymology

Genus, rhizo (G) for “root” and *Smilodon* for the ancestral relationship to the genus. Species, after Barbara Fite, who donated the paratype.

#### Holotype

UF 124634, partial right ramus including mandibular symphysis, c1, m1, and alveoli for p3 and p4 (Figure 1D-F).

#### Paratype

UF 135626, partial left ramus containing p3-m1 (Figure 1G-I).

#### Referred Specimens

UF 22890, partial left ramus containing p3, p4, and the anterior portion of m1; UF 223796 (cast), partial right ramus containing p4-m1; UF 212381, portion of right mandibular symphysis including partial alveoli for c1 and p3; UF 272337 (cast), partial right ramus with p4-m1; UF 65686, distal end of a right humerus; UF 133938, complete left humerus; UF 123836, distal left radius; UF 133939, complete left tibia; UF 212380, right proximal tibia. Dental measurements in Table 3.

**Table 3:** Basic measurements of types and referred dental elements (in mm).

Specimen	p3-m1 Alveolar Lgth.	Diast.	c L	c W	p3L	p3AW	p3 PW	p4 L	p4 AW	p4 PW	m1 L	m1 W
UF 124634	52.3	23.3	14.5	9.2	–	–	–	–	–	–	21.5	9.9
UF 135626	–	–	–	–	11.3	4.8	5.9	18.8	6.9	9.0	22.5	10.4
UF 22890	50.9	–	–	–	12.2	4.6	5.8	18.9	6.6	8.3	–	9.9
UF 232796*	–	–	–	–	–	–	–	19.7	6.0	8.1	21.5	9.0
UF 272337*	–	–	–	–	–	–	–	20.3	7.6	8.8	23.0	10.2

* Cast.

#### Type Locality

The holotype and paratype (UF 124634 and 135626), as well as UF 123836, UF 133938, UF 133939, UF 212380, and UF 212381 were all collected from UF locality PO054, Whidden Creek (Table 1), Fort Meade Mine of the Gardinier Inc., Polk County, Florida; 27.753° N; 81.964° W; upper Bone Valley Formation, latest Hemphillian (Hh4) North American Land Mammal Age (see Appendix S3 for additional description).

#### Diagnosis

Nearly complete verticalization of mandibular symphysis; presence of a weak, but clear mandibular flange; suggestion of crenulations on at least lower canines; lower canine remains large but is moderately compressed laterally; non-procumbent incisor arcade; incisors small; P3 and P4 not aligned (implied by offset of p4 and m1); lack of p2; p3 elongate, but smaller than p4 (<2/3 length); posterior lean of p3 and p4 towards m1; lack of anterior accessory cusp on p3; presence of a small posterior accessory cusp on p3; anterior accessory cusp on p4 small; p3 and p4 off-set (not in straight line); p4 long and blade-like, yet with distal widening around posterior accessory cusp; p4 and m1 also off-set; m1 talonid variably present as small accessory cuspid to slight raised bump at the base of the tooth, never fully formed metaconid; m1 robust as in *Smilodon* with widest point at or anterior to the carnassial notch; and m1 paraconid remains shorter than protoconid.

#### Body mass estimates

**Figure 3:**
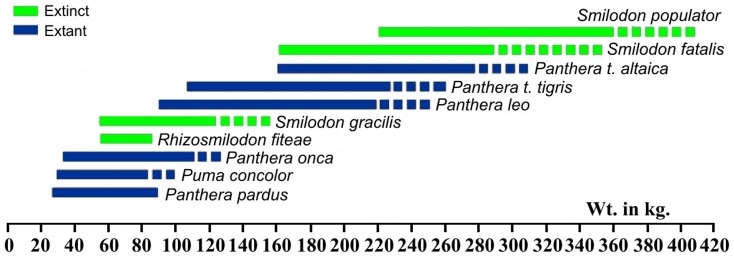
Estimated mass of *Rhizosmilodon fiteae*, following the regression equations of, and modified from, Christiansen and Harris [21], based on the referred humerus and tibia.

Following Christiansen and Harris [21], anterior-posterior diameters (APD) for the referred humerus (UF 133938) and tibia (UF 133939) of 26.3 mm and 18.6 mm, generated body mass estimates of between 76.6–85.0 kg. and 55.7–58.3 kg. respectively (Figure 3). These estimates are similar to observed body masses in extant medium-sized felids such as *Puma concolor* and *Panthera onca*, and also overlap the lower half of the estimated range of *Smilodon gracilis*.

#### Humeri

Though not directly associated with any of the cranial material, the two isolated humeri are here referred to the *R. fiteae* for the following reasons: presence of medial projection of the epicondylar region and straight pectoral ridge (as opposed to strong to sharp distal curve prior to joining the deltoid ridge typical of most pantherine cats, [14]), suggests machairodont affinities. Moreover, the strong curve to the shaft and overall robustness; exaggerated medial projection of the epicondylar region well beyond the entepicondylar foramen, typical of members of *Smilodon* [14], [22]; and thin and elongate wall of bone closing the entepicondylar foramen [14] supports its inclusion within the Smilodontini. Consequently, either there is yet another unknown smilodontine machairodont cat in the Palmetto Fauna, or these humeri do indeed represent the taxon described here. As all medium-sized felid dental remains in the Palmetto Fauna appear to represent a single taxon, *R. fiteae*, all felid post-cranial elements whose size corresponds to these teeth and jaws are also referred to *R. fiteae*.

#### Tree

Notable characters grouping Machairodontini: exaggerated serrations on all teeth; straightening and verticalization of the mandibular symphysis; alignment of P3 with P4 and p4 with m1; elongate and trench-like m1; elongate paraconid (approximately equal to protoconid); slight posterior “lean” to p3/p4. For Smilodontini, including *R. fiteae*: serrations restricted to canines and not as large as in Machairodontini; offset of P3 with P4 and p4 with m1; complete verticalization of mandibular symphysis; m1 shortened and robust with widest point anterior to notch; extreme posterior “lean” to p3/p4. In sum, *R. fiteae* lies basal within Smilodontini (in accord with its older geologic age) and is distinct from taxa within both *Megantereon* and *Smilodon*. Like other members of the Smilodontini: serrations restricted to canines and not as large as in Machairodontini; offset of P3 with P4 implied by that of p4 with m1; Less derived features not typical of more advanced machairodonts, yet present in *R. fiteae*: small anterior and posterior accessory cusps on p4; retention of a large lower canine; and small, non-procumbent incisors.

*Rhizosmilodon fiteae* differs from other members of Smilodontini in polarity depending on the feature. Specifically:

Canine serrations appear to have been very minor (if even present) in *R. fiteae* (specimen worn) and *S. gracilis.* Clear serrations are exhibited by *S. populator* and *S. fatalis*, but apparently are lost in species of *Megantereon.* It should be noted that *S. gracilis* exhibits perhaps the beginning of serrations with upper canines characterized by extreme flattening and elongation, with thin enamel ridge running along the anterior and posterior margins of the tooth. Light crenulations (alternating thick and thin areas) are visible along these ridges on several nearly unworn upper canines of *S. gracilis* (e.g., UF 84189, 86843), – perhaps the precursor to true serrations. Moreover, the recently reported middle Pleistocene record of *Smilodon* from Venezuela [19] was described as possessing fine serrations on the upper canine, yet also exhibiting morphology otherwise more consistent with *S. gracilis*, as opposed to *S. fatalis*. Should this record be correct, then it supports the possibility of serrations or crenulations emerging within Smilodontini in, or close to, *R. fiteae*. Earlier suggestions of *Megantereon* as ancestral to *Smilodon* [11] suffered with the issue of the former lacking serrations, yet some earlier machairodonts exhibiting them (hence representing the acquisition, loss, then re-acquisition of serrations). Evenly spaced valleys and associated ridges, which are nearly equally spaced, are visible on the lower canine of the *R. fiteae*; however wear obscures definitive confirmation of true crenulations. If *R. fiteae* did indeed exhibit very minor serrations or crenulations, the loss of such features in species of *Megatereon*, and contemporaneous development of them in species of *Smilodon*, is not as problematic.

The large size of the lower canine and only moderate development of the mandibular flange suggest a “saber-tooth” configuration more pronounced than species within *Promegantereon* or *Paramachaerodus*; similar to species within *Machairodus*, where the canine is flattened and elongate; but not as extreme as the various species of *Smilodon* or *Megantereon*. Moreover, *R. fiteae* is primitive in its retention of a fairly large lower canine (incisiform in nearly all later machairodonts). However, the lower canine is noticeably laterally compressed. Because enlarged and laterally compressed upper canines, with reduced but also compressed lower canines, are typical machairodont traits [23], [24], taken as a whole the canine configuration exhibited by the *R. fiteae* can be interpreted as a truly intermediate state.

Typical of advanced machairodonts is the protrusion of the incisors into a strong arch [23]–[25]. Limited space between the outer edge of the jaw and the suture at the mandibular symphysis strongly suggests the retention of very small incisors (primitive among machairodonts) in *R. fiteae*. Species of *Megentereon* and *S. gracilis*, exhibit enlarged incisors with the minor development of an arch, which would be intermediate between those of *R. fiteae* and later more advanced machairodonts (e.g. *S. fatalis*, *S. populator* or *Homotherium*).

Many of the major differences between *R. fiteae* and other members of the tribe Smilodontini are exhibited by the lower premolars. Specifically, the distal cusp on the p3 is well developed in *R. fiteae*. On *S. gracilis* it is typically present, but is reduced and in some specimens occurs as a series of tiny accessory cusps. Species of *Megantereon*, typically exhibit the cusp, but also in a greatly reduced state. In *S. fatalis* and *S. populator*, the entire tooth is typically lost. On the p4 of *R. fitaea*, the anterior and distal accessory cusps remain low and small. These are enlarged (proportionally more similar in size to the primary central cusp) in later, more derived machairodonts. In addition, the p4 of *R. fiteae* and some *Megantereon* spp. is somewhat long and blade-like with slight widening at the posterior end of the tooth. In species of *Smilodon* (particularly the later forms), the anterior portion widens as well (also exhibits a larger and more prominent anterior accessory cusp) resulting in an overall more robust tooth.

The lower first molar of *R. fiteae* is also somewhat intermediate in exhibiting a slight hint of a talonid (metaconid). More primitive machairodonts, such as *Promegantereon ogygia* or *Machairodus aphanistus*, typically retain a well-developed metaconid, while the more advanced forms exhibit no evidence of it. In addition, *R. fiteae* retains a simple m1, whereas some advanced members of the genus *Smilodon* (*S. fatalis* and *S. populator*), develop anterior accessory cusps on the m1 (∼ parastylids?).

#### Former identifications

Though several authors have suggested that the original identification of the Palmetto Fauna machairodont as *Megantereon hesperus* [7] was likely incorrect, few offered (supported) alternative identifications. However, Turner [10] expressed two possibilities based on the material known at that time. First was that the taxon could represent a species of the feline-like machairodont *Dinofelis*, similar to that observed in South Africa from similar-aged deposits. Presence of the mandibular flange, flattening of the canines, and potential presence of serrations, in addition to lack of characters typical of the *Dinofelis* [26], now eliminates that taxon. Alternatively, the Palmetto Fauna machairodont could represent *Paramachaerodus* [10], an early machairodont known to exhibit less derived features. Adding to this, Hodnett [13] described new felid material from Arizona (White Cone specimen) as “*Paramachairodus*” sp. and noted its similarity to the Palmetto Fauna machairodont. However, he described the White Cone specimen as gracile and laterally compressed, whereas the mandibles of *R. fiteae* are stout and robust for their length. In addition, the thin ridge of bone on the dorsal surface of the ramus between p3 and c1 described on the White Cone specimen [13] is more strongly developed in *R. fiteae*, but is known to be highly variable among machairodont taxa, so likely carries little taxonomic value [10]. Moreover, the diastema itself is significantly longer and ventrally directed in *R. fiteae*. The White Cone specimen is also described as having a rounded anterior margin [13], whereas *R. fiteae* has a moderately developed, yet clear mandibular flange. A small anterior cuspid described on the p3 of the White Cone specimen is lacking in *R. fiteae*, and the p3 of the former is proportionately larger, relative to the p4, than in the latter. Lastly, the p4 of the White Cone specimen is proportionately smaller than m1 relative to *R. fiteae*. Taken together, the characters describing the White Cone specimen highlight its distinctiveness from *R. fiteae*.

#### Biogeographic implications

The first definitive *Megantereon* in North America occurs in the Blancan [10], [27]. Consequently, our results support two possible evolutionary scenarios: First, the genus *Megantereon* originated in the New World [7], [8], at about the same time (early Blancan) as *Smilodon*, followed by dispersal to, and diversification in, the Old World. *Smilodon* then spreads and diversifies in the New World through the Pliocene and Pleistocene. Morphologic similarities of both genera (particularly between *S. gracilis* and *M. hesperus*) imply similar lifestyles and paleoecology (see Appendix S3 for additional comments). However, geographic separation allowed each genus to thrive and diversify, while avoiding competition with the other. Moreover, the limited NA records of the genus *Megantereon* likely represent last holdovers before it was out-competed by members of the genus *Smilodon*.

Alternately, *R. fiteae* and the species within *Smilodon* do have some features in common to the exclusion of species of *Megantereon* (e.g., the reduced development of the mandibular flange, and persistence of a minor talonid on m1). Consequently, additional characters may amplify the distinction of *Megantereon* from the remainder of the tribe, suggesting a common ancestor of *Megantereon* and its sister taxon: *Smilodon* + *R. fiteae*. If true, such a relationship would then support an Old World origin of *Megantereon* and the tribe Smilodontini [10], [11] and two dispersals of this tribe into NA, once in the Hemphillian, and then again in the Blancan.

## Conclusion

The smaller Palmetto Fauna machairodont does not represent *Megantereon hesperus*; however, it is basal within the tribe Smilodontini, which also includes the genera *Megantereon* and *Smilodon*. Moreover, the unique combination of both conservative and derived characters warrants erection of a new genus and species, *Rhizosmilodon fiteae*. This very late Hemphillian record is the oldest for the tribe, thereby supporting a North American origin at least 5 million years ago (latest Miocene). More specifically, our results also support suggestions of a North American origin of *Smilodon* [16] from a common ancestor with *Megantereon*; also affirming their relationship as sister taxa [7], [15], [17], [18], [28]. Our results refute the suggestion that the Palmetto Fauna machairodont is congeneric with “*Paramachairodus*” (now *Promegantereon* [20]) from Spain [13]. Both *Promegantereon* and *Paramachaerodus* exhibit less derived features than *R. fiteae* and consequently fall distinct from, and more basal to, the Smilodontini. Lastly, *R. fiteae* differs significantly from the White Cone (Arizona) specimen identified as “*Paramachairodus*” sp. [13], consequently the two taxa are not conspecific.

## Supporting Information

Appendix S1 **Character used in this analysis (1-25 modified from** **[20]** **).**
(DOC)

Appendix S2 **Second most parsimonious tree.** Note that the only two taxa that move (compared to the first tree) are the two species of *Paramachaerodus*. TL  =  89, CI  =  0.63, RI  =  0.74, and RC  =  0.47.
(DOC)

Appendix S3 **Supplemental text.**
(DOC)
