# Supplementary material for: A New Machairodont from the Palmetto Fauna (Early Pliocene) of Florida, with Comments on the Origin of the Smilodontini (Mammalia, Carnivora, Felidae)
Source: PLoS One. 2013 Mar 13;8(3):e56173. doi: 10.1371/journal.pone.0056173 (PMC3596359; doi:10.1371/journal.pone.0056173)
Supplement: Appendix S1 — Character used in this analysis (1-25 modified from [20] ). (DOC) [file pone.0056173.s001.doc]

Appendix S1

Character used in this analysis (1-25 modified from [20]).

1. Nasal bones shape. Several species of derived Machairodontinae show wide, rectangular shaped nasal bones. 0) sub-rectangular, relatively narrow. 1) somewhat rectangular, relatively wide. 2) greatly elongate nasals (nearly parallel)
2. Exaggerated serrations 0) absent, or only present on canines 1) occur on virtually all teeth
3. Upper canine crow. The lateral flattening of the upper canines is a derived character of Machairodontinae, but it shows different degree within the different lineages. 0) moderately flattened. 1) markedly flattened.
4. Upper canine crenulations/serrations. This character is present in the most recent Machairodontinae, but also in other, more primitive species, and absent in relatively derived forms. 0) lacking 1) minor crenulations 2) clear serration, but small 3) large and very coarse
5. Upper and lower canines size. The enlargement of the upper canines in the Machairodontinae is linked to a reduction in the size of the lower canines – also can be implied from the presence of a lower flange on dentary. 0) similarly sized. 1) upper canines larger that the lower canines (2-3 times longer) 2) upper canines exaggerated = significantly larger than lower canines (more than 5 times the length)
6. Lower canine 0) large 1) reduced in size, incisiform
7. Upper incisors arcade. Other character present in the more derived Machairodontinae is the development of a procumbent incisor arcade, probably related to the specialized hunting technique of these animals. 0) non-procumbent arcade. 1) procumbent arcade 2) exaggerated procumbent arcade.
8. Lower incisor size 0) significantly smaller than canine 1) large and caniniform (similar in size to the canine)
9. P1 or P2. Generally reduced in Felidae, with P1 and or P2 being present in the more primitive forms. 0) either present. 1) both absent.
10. Orientation of P3 and P4. 0) aligned. 1) not aligned.
11. Anterior cusp of P3. 0) absent. 1) present.
12. P3, disto-lingual basal expansion of crown. The reduction of this basal expansion produces a more trenchant P3, a derived character observed in different lineages of Machairodontinae. 0) present. 1) absent.
13. P3, mesio-distal elongation of crown. The primitive morphology of the P3 shows a relatively short and high crown; this morphology derives in different lineages of Machairodontinae, producing an elongated and relatively low P3. 0) absent. 1) present.
14. Relative size P4-P3. The presence of a relatively large P4 is a derived trait observed in the most recent Machairodontinae. For this character we calculated an index between P4 and P3 length. 0) similar in size. 1) P3 reduced, but still blade like 2) P3 significantly smaller.
15. P4 protocone. The protocone of P4 is placed near the mesial border of parastyle in primitive felids. 0) protocone of P4 placed at the level of the mesial border of the parastyle. 1) protocone of P4 placed distally to the parastyle.
16. P4 protocone 0) still well developed and prominent 1) reduced in size increasing the shearing ability of the blade
17. P4 ectostyle. The presence of an ectostyle in P4 is a derived character present in some very derived Machairodontinae. 0) absent. 1) present.
18. P4 parastyle. The parastyle of P4 is small in primitive species of Machairodontinae and most of the Felinae. 0) smaller/similarly sized than the protocone. 1) clearly larger than the protocone.
19. M1 crown. This tooth is very reduced in Felidae when comparing to other pieces. This reduction is even more extreme in some derived Machairodontinae. 0) bucco-lingually elongated. 1) rounded.
20. Mandibular symphysis outline. The verticalisation of the mandibular symphysis is observed in the more derived species of Machairodontinae, but some primitive species retain a primitive, curved outline. 0) gently curved. 1) straight, but not completely verticalized 2) nearly completely verticalized.
21. Mandibular flange. The development of a mandibular flange is observed in derived Machairodontinae, but some species only show the verticalisation of the mandibular symphysis. 0) absent. 1) present, but small. 2) present, but exaggerated.
22. p2. This tooth is generally absent in Machairodontinae, but it is still present in the more primitive forms 0) present. 1) absent.
23. p3 anterior cuspid. 0) present, small.1) enlarged to prominent cusp 2), absent.
24. p3 distal cuspid. 0) present, small 1) enlarged to prominent cusp 2), absent.
25. p3 0) remains simple 1) elongate, blade like often nearly as long as p4 2) generally reduced (1/2 length or less) 3) very small and peg-like or lost
26. p4 anterior and distal cuspid. The presence of a well-developed mesial cuspid on p4 is a derived character for Felidae. 0) small, minor accessory cusps. 1) well-developed and more prominent.
27. Relative position of p3 and p4. The alignment of p3 and p4 is a primitive character for Felidae. 0) aligned. 1) not aligned.
28. Alignment of m1 and p4. 0) fairly straight. 1) off set, taken in combination with p3-p4 making strongly curved arcade in some taxa.
29. m1 talonid. The talonid in m1 is present, although reduced, in most of the primitive forms of Machairodontinae, and it is lost in the most derived species. 0) always present with well-developed cusp (metaconid) 1) reduced to nearly absent, but hint of talonid. 2) absent.
30. Height of coronoid process. This process is reduced in advanced forms of Machairodonts.0) remains tall as in many extant felids. 1) reduced, but still nearly as tall as ramus is deep. 2) significantly reduced.
31. Robustness of m1 0) blade like, yet not elongate 1) very thin and elongate 2) short and robust
32. p4 0) short, but somewhat blade like 1) very elongate, very blade like 2) somewhat blade like, but with distal widening of the tooth 3) relatively long, but wide and robust
33. m1 parastylid development 0) lacking 1) varies from minor accessory cusp, to fully formed cusp with valley separating it from paraconid
34. m1 width 0) paraconid and protoconid roughly same width on either side of notch 1) widest point of tooth well anterior to notch in paraconid with protoconid significantly tapering back
35. m1 paraconid length. 0) significantly shorter than protoconid. 1) becoming elongate, nearly equal in length.
36. p3 posterior “lean” 0) virtually nonexistent. 1) slight. 2) extreme.
37. p4 posterior “lean” 0) virtually nonexistent. 1) slight. 2) extreme.
