# Supplementary material for: A New Machairodont from the Palmetto Fauna (Early Pliocene) of Florida, with Comments on the Origin of the Smilodontini (Mammalia, Carnivora, Felidae)
Source: PLoS One. 2013 Mar 13;8(3):e56173. doi: 10.1371/journal.pone.0056173 (PMC3596359; doi:10.1371/journal.pone.0056173)

Appendix S2

Second most parsimonious tree. Note that the only two taxa that move (compared to the first tree) are the two species of *Paramachaerodus*. TL = 89, CI = 0.63, RI = 0.74, and RC = 0.47.


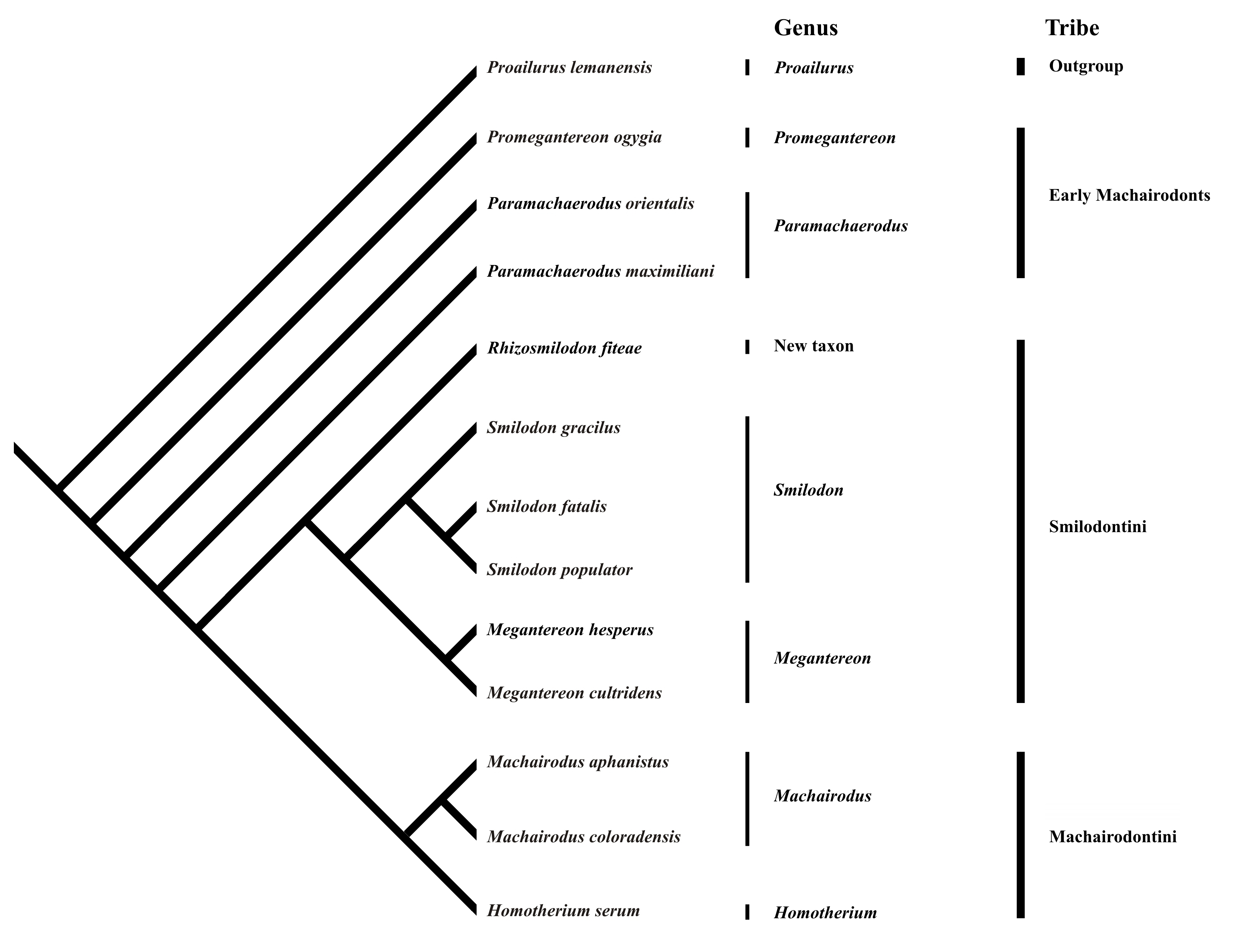

Supplement: Appendix S2 — Second most parsimonious tree. Note that the only two taxa that move (compared to the first tree) are the two species of Paramachaerodus. TL = 89, CI = 0.63, RI = 0.74, and RC = 0.47. (DOC) [file pone.0056173.s002.doc]
