# Supplementary material for: A New Machairodont from the Palmetto Fauna (Early Pliocene) of Florida, with Comments on the Origin of the Smilodontini (Mammalia, Carnivora, Felidae)
Source: PLoS One. 2013 Mar 13;8(3):e56173. doi: 10.1371/journal.pone.0056173 (PMC3596359; doi:10.1371/journal.pone.0056173)
Supplement: Appendix S3 — Supplemental text. (DOC) [file pone.0056173.s003.doc]

**Appendix S3. Supplemental text.**

*Upper Bone Valley Formation* – The Central Florida Phosphate District is an area of several hundred square kilometers covering southwestern Polk County and extending south- and westward into the adjacent counties [1]. Commercial phosphate mining in the district over the past 115 years has created temporary exposures of the richly fossiliferous Mio-Pliocene Bone Valley Formation that is otherwise covered by Quaternary sands and clays. The Bone Valley Formation is extremely varied lithologically, ranging from beds of pure clay to lenses and channel fills of coarse gravel [2]. Both marine and terrestrial vertebrate fossils are frequently recovered in the same horizon. Unlike the Upper Bone Valley Formation, the Lower Bone Valley Formation consists predominantly of beds of clay and sand, with less of a gravel component. Vertebrate biochronology dates the Lower Bone Valley Formation as middle to early late Miocene (early Barstovian–early Hemphillian), while the Upper Bone Valley Formation is very early Pliocene (latest Hemphillian or Hh4) [3, 4, 5]. Over 50 species of reptiles, birds, and mammals have been named on the basis of Bone Valley Formation fossils. The composite vertebrate fossil assemblage from the Upper Bone Valley Formation is referred to as the Palmetto Fauna [3, 6].

*Inferred behavior based on morphology* – Limb strength and/or proportions have already been suggested to imply ambush hunting for at least *S. fatalis* [22, 31, 32]. In addition, several authors [9, 11] have pointed out that the stout front limbs for immobilizing prey, coupled with a specialized killing bite to the throat, suggest a very unique type of hunting for *Megantereon*, most likely ambush style with a rapid “kill bite”. Such features appear to have evolved in parallel within machairodonts, with the Smilodontini simply taking both features to an almost ridiculous extreme. Specifically, simulations using *S. fatalis* [33] support a rapid, efficient kill bit primarily driven through neck muscles. Similar conclusions have also been reached for *Homotherium latidens* [34] and *Machairodus aphanistus* [23]. Therrien [35] and Christiansen [36] also showed the significantly stronger bite force exhibited by saber-toothed cats (relative to pantherine cats). It should be noted however, that robust forelimbs and elongated canines did originate prior to this exaggeration, and that many transitional forms document the evolution of these features. For example, Salesa et al [27] noted the development of large forelimbs and robust “thumb” claw in *Promegantereon ogygia* from the late Miocene of Spain, suggesting importance of rapidly immobilizing prey (also see [11, 24, 34, 38] for similar conclusions for different taxa), as opposed to the ability to capture larger prey. Meachen-Samuels and Van Valkenburgh [39] highlighted the culmination of such strong forearms in *S. fatalis*, hypothesizing the ability to rapidly immobilize prey was primarily to reduce the possibility of damaging the elongate canines as a result of struggling prey. However, it seems that these two structures were evolving simultaneously as part of an overall rapid-kill system.

Although traditional interpretations of taxa like *S. fatalis* have been that of a carnivoran specialized for hunting larger prey (e.g., [36, 40] and citations therein), Andersson et al [41] suggested, based on mechanical and physiological constraints, that taxa within *Smilodon* and *Megantereon* were actually better suited for killing prey of similar, or smaller, body size. Feranec [42] recovered isotopic values from the tooth enamel of *S. gracilis* from Leisey Shell Pit 1A locality in Florida, that implied it was feeding on C_3_, rather than C_4_, consumers. Although Feranec [42] suggested *Platygonus* and *Hemiauchenia* as likely prey (as opposed to *Equus* or *Mammuthus*, both common C_4_ consumers at the site), the cervid *Odocoileus* would also be consistent with such values. Similar isotopic results on *Megantereon* *whitei* from Europe were specifically interpreted to be the result of consuming cervids [43]. Moreover, isotopic values from teeth of *S. fatalis* suggest that it was consuming prey feeding in marginal areas between woodland and grassland habitats in South Carolina [44], and prey eating primarily C_3_ plants at La Brea [45]. Neither study directly suggests feeding on cervids, but neither precludes it. Because *R. fiteae* exhibits the early indications of this highly modified and specialized morphology towards prey immobilization coupled with a rapid “kill bite”, it seems entirely possible that this taxon may represent the origins of this style of hunting, which persisted until the end of the Pleistocene with the extinction of *Smilodon* spp. If the Andersson et al [41] hypothesis is true, the Palmetto Fauna includes numerous small- to medium-size ungulates that would have been optimal-sized prey for *R. fiteae*, including two small hipparionine equids (*Nannippus aztecus* and *Pseudhipparion simpsoni*), a small tapir (*Tapirus polkensis*), two tayassuids (*Mylohyus elmorei* and *Catagonus brachydontus*), a small lamine (*Hemiauchenia edensis*), a small hornless ruminant (*Floridameryx floridanus*), two antilocaprids (*Subantilocapra garciae* and *Hexameryx simpsoni*), and a cervid (*Eocoileus* *gentryorum*). It is intriguing that the first known appearances of both the Smilodontini and Cervidae in North America are in the Palmetto Fauna of Florida.

Fortuitously, the first appearance of Cervidae in North America, *Eocoileus* from Florida [46], during the late Hemphillian, and the fact that cervids are very abundant at the type locality of *R. fiteae* (Whidden Creek), suggest the evolution of the Smilodontini in southeastern North America at that time to exploit such prey. Moreover, the exaggerated features of Smilodontini were selected specifically to hunt and kill similar sized (or smaller) prey, as suggested for machairodonts in general [37, 41]. It’s also been further suggested [24, 37] that specialized postcrania within machairodonts was first to increase efficiency for immobilizing prey, and that the extreme modification of the upper canines came much later within the group to take advantage of such prey with a rapid and very efficient “kill bite”.
